# Long-Term Hepatocellular Carcinoma Development and Predictive Ability of Non-Invasive Scoring Systems in Patients with HCV-Related Cirrhosis Treated with Direct-Acting Antivirals

**DOI:** 10.3390/cancers14030828

**Published:** 2022-02-06

**Authors:** Gian Paolo Caviglia, Giulia Troshina, Umberto Santaniello, Giulia Rosati, Francesco Bombaci, Giovanni Birolo, Aurora Nicolosi, Giorgio Maria Saracco, Alessia Ciancio

**Affiliations:** 1 Liver Unit, Department of Medical Sciences, University of Torino, 10126 Turin, Italy; giulia.troshina@gmail.com (G.T.); umberto.santaniello@edu.unito.it (U.S.); giulia.rosati@edu.unito.it (G.R.); francesco.bombaci@edu.unito.it (F.B.); giovanni-birolo@unito.it (G.B.); aurora.nicolosi@unito.it (A.N.); giorgiomaria.saracco@unito.it (G.M.S.); 2 Gastroenterology Unit, Department of General and Specialistic Medicine, A.O.U. Città della Salute e della Scienza—Molinette Hospital, 10126 Turin, Italy

**Keywords:** ALBI, DAA, FIB-4, HCC, HCV, NSS

## Abstract

**Simple Summary:**

In the present study we investigated the ability of different non-invasive scoring systems (i.e., Forns index, APRI, FIB-4, ALBI, and aMAP) to predict hepatocellular carcinoma development in a large cohort of patients with HCV-related cirrhosis treated with direct-acting antivirals followed for a median of 44.9 months. ALBI score showed the best performance for the prediction of hepatocellular carcinoma; this score may be a useful inexpensive tool for risk stratification and the personalization of hepatocellular carcinoma surveillance strategy for patients with cirrhosis and previous history of HCV infection treated with DAA.

**Abstract:**

Patients with hepatitis C virus (HCV)-related cirrhosis treated with direct-acting antivirals (DAA) are still at risk of developing hepatocellular carcinoma (HCC). We investigated the accuracy of non-invasive scoring systems (NSS) for the prediction of de novo HCC development in patients treated with DAA on long-term follow-up (FU). We analyzed data from 575 consecutive patients with cirrhosis and no history of HCC who achieved a sustained virologic response (SVR) to DAA therapy. NSS (i.e., Forns index, APRI, FIB-4, ALBI, and aMAP) were calculated at 3 months after the end of therapy. Performance for de novo HCC prediction was evaluated in terms of area under the curve (AUC) and Harrell’s C-index. During a median FU of 44.9 (27.8–58.6) months, 57 (9.9%) patients developed de novo HCC. All five NSS were associated with the risk of de novo HCC. At multivariate analysis, only the ALBI score resulted in being significantly and independently associated with de novo HCC development (adjusted hazard ratio = 4.91, 95% CI 2.91–8.28, *p* < 0.001). ALBI showed the highest diagnostic accuracy for the detection of de novo HCC at 1-, 3-, and 5-years of FU, with AUC values of 0.81 (95% CI 0.78–0.85), 0.71 (95% CI 0.66–0.75), and 0.68 (95% CI 0.59–0.76), respectively. Consistently, the best predictive performance assessed by Harrell’s C-statistic was observed for ALBI (C-index = 0.70, 95% CI 0.62–0.77). ALBI score may represent a valuable and inexpensive tool for risk stratification and the personalization of an HCC surveillance strategy for patients with cirrhosis and previous history of HCV infection treated with DAA.

## 1. Introduction

Hepatocellular carcinoma (HCC) accounts for more than 90% of primary liver cancers and represents a major health problem worldwide, with 841,000 new cases and 782,000 deaths per year [1,2]. In western countries, the largest fraction of HCC is attributable to chronic hepatitis C virus (HCV) infection [3]. However, irrespective of the underlying liver disease etiology, cirrhosis is the principal risk factor for HCC development [4]; even following hepatitis C virus (HCV) eradication by direct-acting antiviral (DAA) treatment [5], patients with cirrhosis post-sustained virologic response (SVR) are still at risk of HCC development [6,7].

Given that an HCV cure reduces but does not eliminate the risk of HCC incidence, all patients with HCV-related cirrhosis should be entered into surveillance programs [8]. According to international guidelines, surveillance should be performed by means of abdominal ultrasound (US) every 6 months, with or without the measurement of alpha-fetoprotein (AFP) [9,10]. However, given the high heterogeneity of HCC, a more personalized surveillance strategy based on individual risk assessment is an unmet need. Promising results have been obtained from non-invasive scoring systems (NSS) combining demographic, clinical, and biochemical variables normally collected during standard clinical practice [11,12]. Due to their cost-effectiveness, this approach could be suitable for the surveillance of patients at risk of HCC development. However, the accuracy of these NSS in predicting HCC occurrence in patients with HCV-related cirrhosis following DAA treatment remains uncertain.

In 2017, a Japanese study compared the utility of three laboratory indices of liver fibrosis, namely aspartate aminotransferase-platelet ratio index (APRI), fibrosis-4 (FIB-4), and Forns index, for the surveillance of patients with chronic HCV infection post-SVR, showing that the Forns index was the most accurate for the identification of patients at low risk of HCC development [13]. More recently, a multicenter Italian study reported that the albumin–bilirubin (ALBI) score, originally developed for liver function evaluation in HCC patients, was independently associated with de novo HCC (HR = 2.35, *p* = 0.038) in patients with HCV-related cirrhosis receiving DAA therapy [14]. Finally, a model combining age, gender, albumin–bilirubin, and platelets (i.e., aMAP score), specifically developed for the prediction of HCC development in patients with chronic hepatitis, showed accuracies from 0.68 to 0.77 in post-SVR cirrhotic patients [15].

In this study, we aimed to investigate and compare the prognostic accuracy of the most common NSS (APRI, FIB-4, Forns index, and ALBI) and of the recently proposed aMAP in a large cohort of patients with HCV-related cirrhosis post-DAA treatment, under surveillance for the risk of de novo HCC development.

## 2. Materials and Methods

### 2.1. Patients

This is a single-center, observational study of consecutive patients with chronic HCV infection who underwent first-line DAA treatment between June 2014 and December 2020 at the Liver Unit of the Department of Medical Sciences, University of Torino, Turin, Italy.

**Figure 1:**
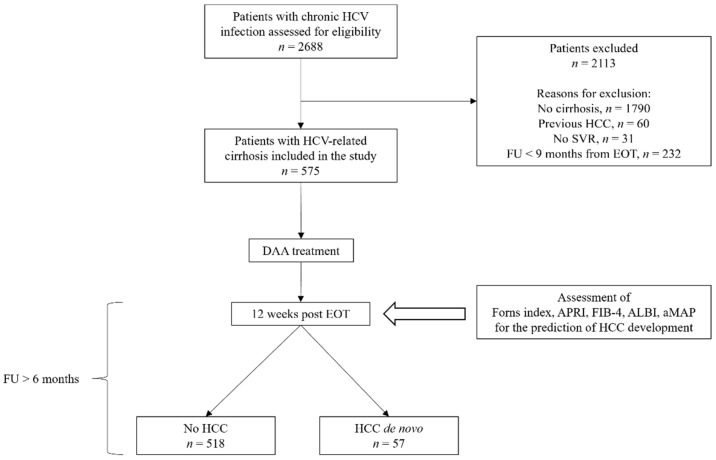
Flow chart of the study population. Abbreviations: direct-acting antivirals (DAA), end of treatment (EOT), follow-up (FU), hepatocellular carcinoma (HCC), hepatitis C virus (HCV), number (*n*), and sustained virologic response (SVR).

Patients were included in the present study if they fulfilled the following criteria: age > 18 years, SVR to DAA treatment, diagnosis of cirrhosis, and at least 9 months of follow-up (FU) after end of treatment (EOT). Exclusion criteria were previous history of HCC, no SVR, testing positive for hepatitis B surface antigen, weekly alcohol consumption > 140 g in women and >210 g in men, and lack of written informed consent. The flow chart of the study is depicted in Figure 1.

Before DAA initiation, a complete medical history and physical examination were performed, and the following data were collected and recorded in a dedicated database: age, gender, body mass index (BMI), risk factors for HCV infection, comorbidities, and previous treatments for chronic HCV infection. Blood samples were drawn for virologic and biochemical investigations that included: HCV RNA (AmpliPrep^®^/COBAS Taqman^®^ HCV test, Roche Diagnostics, Basel, Switzerland), HCV genotype (VERSANT^®^ HCV Genotype 2.0 Assay Line Probe Assay, Siemens Healthcare Siemens Healthcare, Tarrytown, NY, USA), complete blood count, alanine aminotransferase (ALT), aspartate aminotransferase (AST), gamma-glutamyl transferase (γGT), albumin, total bilirubin, cholesterol, triglycerides, and creatinine. At each further visit, medical history was reviewed along with a routine laboratory work-up, while the US surveillance at a 6-month schedule was not performed at our clinic but was the responsibility of the general practitioners.

SVR was defined as the clearance of HCV RNA at 12 weeks after EOT [16]. Treatment failure was defined as no SVR at 12 weeks of FU due to primary non-response (lack of undetectable HCV RNA during treatment), virologic breakthrough (detectable HCV RNA at EOT following undetectable HCV RNA in course of treatment), or relapse (undetectable HCV RNA at EOT but detectable HCV RNA at 12 weeks of FU).

The diagnosis of liver cirrhosis was achieved by liver biopsy, or by liver elastography (FibroScan^®^, Echosens™, Paris, France) showing liver stiffness measurement > 11.9 kPa [17], and/or by hepatic ultrasound features (i.e., blunted nodular liver edges) and endoscopic/clinical signs of portal hypertension [18]. The diagnosis of HCC was established by histological examination or by contrast-enhanced imaging methods showing hypervascularity in late arterial phase and washout on portal venous and/or delayed phases [9]. HCC was classified according to the Barcelona Clinic Liver Cancer (BCLC) staging system (0 = very early; A = early; B = intermediate; C = advanced; and D = end-stage) [9].

### 2.2. Non-Invasive Scores

We assessed the predictive value of the following 5 NSS, calculated at 12 weeks of FU after EOT according to their originally reported formula:

Forns index [19]: 7.811 − 3.131 * ln Platelets (10^9^/L) + 0.781 * ln γGT (U/L) + 3.467 ln Age (years) − 0.014 * Cholesterol (mg/dL)
APRI [20]: (AST/AST upper limit normal)/Platelets (10^9^/L)) * 100
FIB-4 [21]: (Age (years) * AST (U/L))/(Platelets (10^9^/L) * √ALT (U/L))
ALBI [22]: (Log Total Bilirubin (µmol/L) * 0.66) + (Albumin (g/L) * −0.085)
aMAP [15]: ((0.06 * Age (years) + 0.89 * Sex (Male: 1; Female: 0) + (0.48 × (Log Total Bilirubin (µmol/L) * 0.66) + (Albumin (g/L) * −0.085)) − 0.01 * Platelets (10^3^/mm^3^) + 7.4)/14.77) * 100

### 2.3. Statistical Analysis

Continuous variables were reported by median and interquartile range (IQR), while categorical variables as number (*n*) and percentage (%). Data normality was checked by the D’Agostino–Pearson test. Comparison between unpaired groups was performed by the Mann–Whitney test for continuous variables and by Fisher’s exact test for categorical variables.

Cut-offs for dichotomization of continuous variables were identified by Yuden J-statistic. Survival analysis was carried out according to the Kaplan–Meier method and survival curves were compared using the log-rank test. Cox proportional hazards regression analysis was used to evaluate the association between NSS and HCC development; the strength of the association was reported as hazard ratio (HR) with the corresponding 95% confidence interval (CI).

Receiver operating characteristic (ROC) curve analysis was performed to assess NSS diagnostic accuracy for HCC detection at 1-, 3-, and 5-years; NSS performance was reported as area under the curve (AUC). Predictiveness was evaluated by Harrell’s concordance index (C-index); the 95% CI have been estimated by bootstrapping 1000 times.

A two-tailed *p* value < 0.05 was considered statistically significant. All the statistical analyses were performed using MedCalc software, version 18.9.1 (MedCalc bvba, Ostend, Belgium) and the scikit-learn (version 0.24.2) and scikit-survival (0.15.0) Python packages.

## 3. Results

### 3.1. Characteristics of the Study Cohort

Overall, 2688 patients with chronic HCV infection who underwent DAA treatment during the study period were assessed for eligibility. After excluding patients without cirrhosis (*n* = 1790), patients with a previous history of HCC (*n* = 60), patients who did not achieve SVR to DAA therapy (*n* = 31), and those at FU < 9 months from EOT (*n* = 232), a total of 575 consecutive patients were included in the study.

Among non-SVR patients (*n* = 31), 8 (25.0%) patients were primary non-responders, 2 (6.3%) experienced a virologic breakthrough, 20 (62.3%) relapsed, and 2 patients discontinued DAA because of severe side effects. Of note, 24 patients were subsequently retreated after first-line DAA and achievied SVR.

During a median FU of 44.9 (27.8–58.6) months, 57 (9.9%) patients developed de novo HCC (BCLC staging: 0, *n* = 11, 19.3%; A, *n* = 26, 45.6%; B, *n* = 9, 15.8%; C, *n* = 11, 19.3%). The median censoring time was 40.6 (22.9–53.4) months, while median time to de novo HCC from 12 weeks of FU after EOT was 29.3 (16.4–41.1) months. The estimated cumulative incidence of HCC at 1-, 3-, 5-years of FU was 2.0% (±0.6%), 9.9% (±1.4%), and 18.7% (±3.5%), respectively.

The demographic, clinical, and biochemical characteristics at 12 weeks of FU after EOT are reported in Table 1. Median age was 65 (57–77) years and most patients were males (*n* = 331; 57.6%), with a slightly high prevalence of males among patients who developed HCC (*p* = 0.048). No other differences were observed regarding demographic and anamnestic features between patients who developed de novo HCC and those who were HCC-free at last FU. Conversely, the former had a more severe liver disease as compared to the latter, showing lower rates of Child–Pugh score A (84.2% vs. 94.2%, *p* < 0.001), lower platelet count (98 × 10^9^/L vs. 130 × 10^9^/L, *p* < 0.001), lower serum albumin values (4.2 g/dL vs. 4.3 g/dL, *p* < 0.001), and higher total bilirubin levels (1.1 mg/dL vs. 0.7 mg/dL, *p* < 0.001). Furthermore, patients who developed de novo HCC had higher rates of cirrhosis complications, including portal hypertension and esophageal varices.

### 3.2. Prediction of HCC Development Using NSS

**Figure 2:**
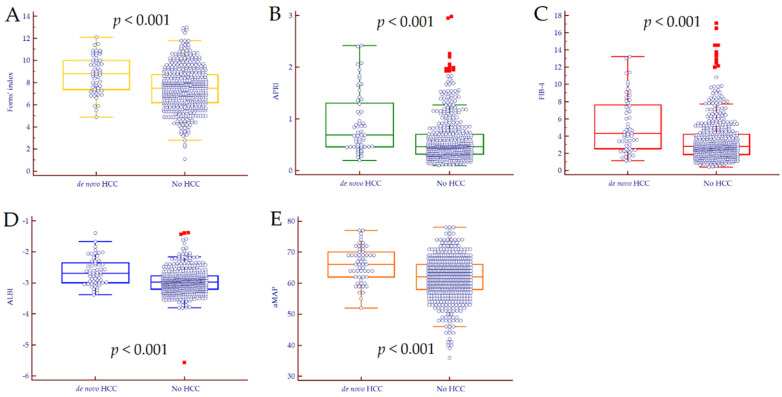
Median values of Forns index (**A**), APRI (**B**), FIB-4 (**C**), ALBI (**D**), and aMAP (**E**) in patients with de novo HCC compared to those HCC-free at last FU. *p* values were calculated by Mann–Whitney test. Abbreviations: hepatocellular carcinoma (HCC).

At 12 weeks after EOT, median NSS values were significantly different between patients who developed de novo HCC and those who did not (Figure 2); respectively, median Forns index was 8.8 (7.4–20.0) vs. 7.5 (6.2–8.7), *p* < 0.001, median APRI values were 0.69 (0.46–1.31) vs. 0.46 (0.32–0.70), *p* < 0.001, median FIB-4 values were 4.34 (2.54–7.61) vs. 2.82 (1.88–4.23), *p* < 0.001, median ALBI score was −2.69 (−2.99–−2.35) vs. −2.98 (−3.20–−2.78), *p* < 0.001, and median aMAP score was 66 (62–70) vs. 62 (58–66), *p* < 0.001.

**Figure 3:**
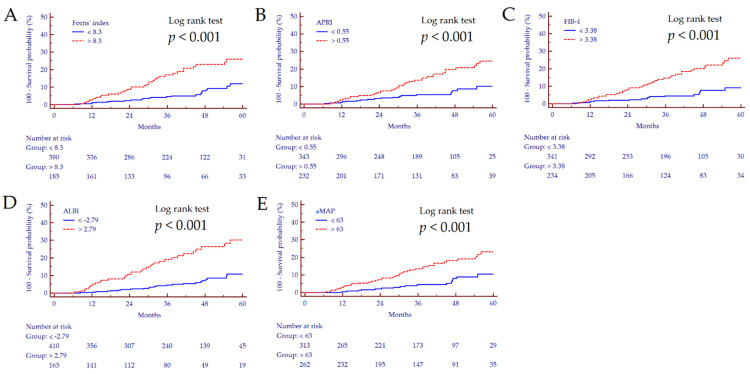
Kaplan–Meier survival curves of patients with cirrhosis followed for the risk of de novo HCC development stratified according to Forns index (**A**), APRI (**B**), FIB-4 (**C**), ALBI (**D**), and aMAP (**E**).

By Yuden J-statistic, we identified the best cut-off values for the dichotomization of NSS values; accordingly, we observed significantly different survival curves for each score (all *p* < 0.001) (Figure 3).

At univariate Cox proportional hazards regression analysis, ALBI > −2.79 showed the strongest association with HCC development (HR = 3.97, 95% CI 2.34–6.74), followed by FIB-4 > 0.55 (HR = 3.37, 95% CI 1.91–5.96), Forns index > 8.3 (HR = 3.02, 95% CI 1.78–5.14), aMAP > 63 (HR = 2.85, 95% CI 1.60–5.09), and lastly by APRI > 0.55 (HR = 2.78, 95% CI 1.60–4.83) (Table 2). However, by stepwise multivariate analysis, ALBI > −2.79 was the only factor significantly associated with HCC development. The same holds true for multivariate analysis that included the five scores as continuous variables; ALBI was the only score that remained significantly associated with HCC occurrence (HR = 4.91, 95% CI 2.91–8.28, *p* < 0.001). Notably, the 1-, 3-, and 5-years estimated the cumulative incidence of de novo HCC for ALBI ≤ −2.79 were 0.5% (±0.4%), 4.4% (±1.2%), and 10.7% (±2.4%) compared to 5.1% (±1.8%), 20.1% (±3.6%), and 30.3% (±4.9%), respectively, for ALBI > −2.79.

**Figure 4:**
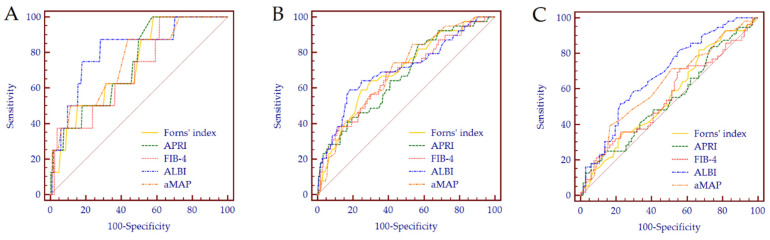
ROC curves of the 5 NSS for the detection of de novo HCC at 1- (**A**), 3- (**B**), and 5-years of FU (**C**).

Among the five NSS, ALBI showed the highest diagnostic accuracy for the detection of HCC at 1-, 3-, and 5-years of FU, with AUC values of 0.81 (95% CI 0.78–0.85), 0.71 (95% CI 0.66–0.75), and 0.68 (95% CI 0.59–0.76), respectively (Figure 4). Consistently, the best predictive performance assessed by Harrel’s C-statistic was observed for ALBI (C-index = 0.70, 95% CI 0.63–0.76); all the other scores achieved a C-index < 0.70 (Table 3).

## 4. Discussion

In the present study, we provided meaningful insights both on the residual risk of de novo HCC development in patients with HCV-related cirrhosis who achieved SVR following DAA treatment and on the performance of five NSS as potential predictors of de novo HCC occurrence during long-term FU post-DAA. Interestingly, we observed that among the Forns index, APRI, FIB-4, ALBI, and aMAP, the best performance for risk stratification and de novo HCC prediction was attained by the ALBI score.

Several studies have shown that in patients with HCV-related cirrhosis successfully treated with DAA, the risk of liver-related events distinctly diminished, particularly in those with preserved liver function [23]. However, the risk of HCC is not completely abolished, but persists overtime, due to HCV-induced epigenetic and gene expression alterations associated to the oncogenic process underlying HCC development [24]. In fact, a recent study by D’Ambrosio and colleagues showed that HCC was the most frequent liver-related event in patients with HCV-related cirrhosis that achieved SVR following DAA, irrespective of liver function status and previous history of liver-related events [25]. In patients with no history of HCC (*n* = 569), the authors observed 46 (8.08%) new HCC cases during a median FU of 51 (range 8–68) months [25]; in our series (*n* = 575) we observed similar results, with a total of 57 (9.9%) patients who developed de novo HCC during a median FU of 44.9 (IQR 27.8–58.6) months. Finally, it is noteworthy that the high prevalence of advanced stage HCC (BCLC B and C) in our cohort (*n* = 25, 38.5%) is possibly linked to the suboptimal rate of retention to the surveillance of SVR patients, due to the false perception that HCV eradication corresponds to the cure of liver disease. Both hepatologists and general practitioners should be aware of this issue and patients should continue to be closely monitored independent of the virologic outcome. In this regard, NSS may represent valuable and inexpensive tools to stratify SVR cirrhotic patients according to their risk of de novo HCC development, and thus implement more personalized surveillance strategies [26]. On the other hand, it should be acknowledged that other studies reported high rates of multinodular HCC or tumors with aggressive patterns occurring in patients treated with DAA as compared to those treated with IFN-based therapy or those who were untreated [27]; it is possible that the perturbation of the immune system induced by HCV eradication following DAA may lead to a rapid tumor evolution from microscopic, undetectable foci of HCC in some cases.

To the best of our knowledge, this is the first study that compared the predictive performance of widely known NSS for de novo HCC development in a large cohort of patients with HCV-related cirrhosis treated with DAA on long-term FU. A direct comparison between the Forns index, APRI, FIB-4, ALBI, and aMAP revealed that ALBI was the only score that remained significantly associated with the risk of HCC development; furthermore, ALBI showed the higher predictiveness, and proved acceptable diagnostic performance especially in the short/medium-term FU. In agreement with our findings, Casadei Gardini and colleagues reported that an increase in ALBI grade (HR = 2.35, *p* = 0.038) was independently associated with HCC development in patients with HCV-related cirrhosis and no history of HCC treated with DAA [14]. Two recent studies from Japan, including patients with advanced fibrosis/cirrhosis who achieved a SVR after DAA treatment, identified ALBI score as an independent factor affecting HCC occurrence in patients with liver cirrhosis [28,29]. In particular, Tanaka and colleagues observed that ALBI, but not FIB-4, was associated with higher HCC risk at multivariate analysis (aHR = 2.5, *p* < 0.001) [29]. Taken together, these results may suggest that NSS reflecting the hepatic function rather than the stage of liver fibrosis, are better predictors for the risk of de novo HCC occurrence.

The study may be limited by its retrospective design; however, all patients were consecutively enrolled, thus providing, in a real-life setting, an unbiased picture of the incidence of de novo HCC in patients with HCV-related cirrhosis treated with DAA with no previous history of HCC. As another possible limitation, we did not assess the performance of other valuable models, such as those that include HCC serological biomarkers as covariates [30]; since biomarkers, including AFP, are not universally recommended for the surveillance of patients at risk of HCC development, we deliberately chose to investigate only NSS, including variables routinely collected during standard clinical practice. Finally, we did not investigate liver steatosis as a potential risk factor for HCC development [31]. Further studies are warranted to specifically assess whether the severity of liver steatosis and metabolic derangements may affect the cure of liver disease and the occurrence of HCC in patients with chronic HCV infection treated with DAA. Of note, our study has several strengths, such as the large number of patients included by a single tertiary-care center and the long FU, that allowed to assess the performance of the NSS, both in the short term after EOT and in the long term, up to approximately 5 years post-DAA treatment.

## 5. Conclusions

In conclusion, in patients with HCV-related cirrhosis who achieved SVR following DAA treatment, we confirmed previous data on the residual risk of de novo HCC development even in the long-term FU. Furthermore, we have provided evidence of the predictive performance for de novo HCC occurrence of NSS widely used in clinical practice. Among the five analyzed NSS, ALBI resulted in the only score significantly and independently associated with the risk of de novo HCC, qualifying as the most accurate non-invasive tool for patients’ surveillance. Further prospective studies are needed to evaluate whether the use of the ALBI score as a diagnostic complement could help clinicians to tailor more individualized surveillance strategies for patients with HCV-related cirrhosis treated with DAA.

## Figures and Tables

**Table 1:** Characteristics of the overall patients enrolled in the study, and according to de novo HCC development.

Characteristics	Overall	No HCC	De Novo HCC	*p*-Value
Patients, *n*	575	518 (81.1%)	57 (9.9%)	
Age (years), median (IQR)	65 (57–77)	65 (57–77)	67 (61–77)	0.193
Gender (M/F), *n*	331/244	291/227	40/17	0.048
Italians, *n* (%) *	537 (93.4%)	482 (93.1%)	55 (96.5%)	0.570
BMI (kg/m^2^), median (IQR)	24.6 (22.7–27.5)	24.6 (22.6–27.6)	24.6 (23.3–27.1)	0.763
T2DM, *n* (%)	108 (18.8%)	99 (19.1%)	9 (15.8%)	0.721
HBV exposure, *n* (%) **	90 (15.7%)	84 (16.2%)	6 (10.5%)	0.338
SOF-based treatment, *n* (%) ^†^	500 (87.0%)	447 (86.3%)	53 (93.0%)	0.212
ALT (U/L), median (IQR)	20 (16–25)	20 (16–25)	22 (18–28)	0.044
AST (U/L), median (IQR)	24 (20–29)	24 (20–29)	30 (24–37)	<0.001
γGT (U/L), median (IQR)	28 (19–40)	28 (18–38)	34 (23–55)	0.001
Platelets (10^9^/L), median (IQR)	130 (92–166)	130 (97–167)	98 (67–138)	<0.001
Albumin (g/dL), median (IQR)	4.3 (4.1–4.5)	4.3 (4.2–4.6)	4.2 (3.8–4.3)	<0.001
Total bilirubin (mg/dL), median (IQR)	0.7 (0.5–1.0)	0.7 (0.5–0.9)	1.1 (0.6–1.7)	<0.001
Cholesterol (mg/dL), median (IQR)	165 (147–185)	165 (148–186)	162 (141–167)	0.020
Triglycerides (mg/dL), median (IQR)	88 (71–111)	88 (72–115)	77 (68–88)	0.007
Child-Pugh score A, *n* (%)	536 (93.2%)	488 (94.2%)	48 (84.2%)	0.010
Portal hypertension, *n* (%)	190 (33.0%)	160 (30.9%)	30 (52.6%)	0.002
Esophageal varices, *n* (%)	153 (26.6%)	125 (24.1%)	28 (49.1%)	<0.001
Ascites, *n* (%)	44 (7.7%)	37 (7.1%)	7 (12.3%)	0.185

* A total of 6 (1.0%) patients were from western Europe, 23 (4.0%) patients were from eastern Europe, 7 (1.2%) patients were from Africa, and 2 (0.3%) patients were from South America. ** Positive for anti-hepatitis B core antibodies. ^†^ 68 (11.8%) patients underwent sofosbuvir + ribavirin, 55 (9.6%) sofosbuvir + daclatasvir ± ribavirin, 253 (44.0%) sofosbuvir + ledipasvir ± ribavirin, 19 (3.3%) sofosbuvir + simeprevir ± ribavirin, 104 (18.1%) sofosbuvir + velpatasvir ± ribavirin, 1 (0.2%) sofosbuvir + velpatasvir + voxilaprevir, 7 (1.2%) elbasvir + grazoprevir, 3 (0.5%) glecaprevir + pibrentasvir, 12 (2.1%) ombitasvir + paritaprevir + ritonavir + ribavirin, and 53 (9.2%) ombitasvir + paritaprevir + ritonavir + dasabuvir ± ribavirin. Continuous variables were compared by Mann–Whitney test, while categorical variables were calculated by Fisher’s exact test. Abbreviations: alanine aminotransferase (ALT), aspartate aminotransferase (AST), body mass index (BMI), female (F), gamma-glutamyl transpeptidase (γGT), hepatitis B virus (HBV), hepatocellular carcinoma (HCC), interquartile range (IQR), male (M), number (*n*), sofosbuvir (SOF), and type 2 diabetes mellitus (T2DM).

**Table 2:** Cumulative incidence of de novo HCC and corresponding hazard ratios according to NSS values.

NSS	Cut-Off	HCC, *n* (%)	*p*-Value *	HR, 95%CI	*p*-Value **
Forns Index	≤8.3 >8.3	23/390 (5.9%) 34/185 (18.4%)	<0.001	3.02, 1.78–5.14	<0.001
APRI	≤0.55 >0.55	19/343 (5.5%) 38/232 (16.4%)	<0.001	2.78, 1.60–4.83	<0.001
FIB-4	≤3.38 >3.38	17/341 (5.0%) 40/234 (17.1%)	<0.001	3.37, 1.91–5.96	<0.001
ALBI	≤−2.79 >−2.79	23/410 (5.6%) 34/165 (20.6%)	<0.001	3.97, 2.34–6.74	<0.001
aMAP	≤63 >63	16/297 (5.1%) 41/262 (15.7%)	<0.001	2.85, 1.60–5.09	<0.001

Cut-off values were calculated by Yuden J-statistic. * calculated by log-rank test. ** calculated by Cox proportional hazards regression analysis. Abbreviations: confidence interval (CI), hazard ratio (HR), hepatocellular carcinoma (HCC), hazard ratio (HR), and non-invasive scoring system (NSS).

**Table 3:** Diagnostic accuracies for the detection of de novo HCC at 1-, 3-, and 5-years of FU, and predictive performance of the 5 NSS.

NSS	1-Year FU AUC (95% CI)	3-Years FU AUC (95% CI)	5-Years FU AUC (95% CI)	C-Index (95% CI)
Forns index	0.73 (0.69–0.77)	0.70 (0.65–0.75)	0.56 (0.47–0.65)	0.68 (0.61–0.75)
APRI	0.74 (0.70–0.78)	0.67 (0.62–0.72)	0.53 (0.45–0.63)	0.67 (0.59–0.74)
FIB-4	0.70 (0.66–0.74)	0.68 (0.63–0.72)	0.55 (0.46–0.64)	0.67 (0.59–0.74)
ALBI	0.81 (0.78–0.85)	0.71 (0.66–0.75)	0.68 (0.59–0.76)	0.70 (0.62–0.77)
aMAP	0.75 (0.71–0.79)	0.70 (0.65–0.74)	0.62 (0.53–0.71)	0.69 (0.62–0.76)

AUC values were calculated by ROC curve analysis, while C-indices were calculated by Harrell’s C statistic. Abbreviations: area under the curve (AUC), confidence interval (CI), concordance index (C-index), follow-up (FU), and non-invasive scoring system (NSS).

## Data Availability

The data presented in this study are available upon request from the corresponding author.
